# Treatment of Atypical Fracture of the Ulnar Diaphysis by Open Reduction and Internal Fixation with Teriparatide

**DOI:** 10.1155/2019/9103412

**Published:** 2019-09-04

**Authors:** Hiroki Ito, Naohisa Miyakoshi, Yuji Kasukawa, Takeshi Sato, Hitoshi Kubota, Hiroshi Sasaki, Takashi Mizutani, Yoichi Shimada

**Affiliations:** ^1^Department of Orthopaedic Surgery, Noshiro Kosei Medical Center, Kamimaedatinai, Noshiro, Akita 016-0014, Japan; ^2^Department of Orthopedic Surgery, Akita University Graduate School of Medicine, 1-1-1 Hondo, Akita 010-8543, Japan

## Abstract

Atypical fractures commonly arise in the subtrochanteric region or the femoral shaft, whereas those of the upper extremities are rare. Only 15 fractures in 13 patients have been described in the English literature. The management of such fractures has not been established. We describe a patient with an atypical fracture of the ulnar diaphysis, which required revision surgery to achieve the union of the fracture site. Teriparatide together with low-intensity pulsed ultrasound contributed to bone healing. Further studies are needed to determine the optimal strategy for treating atypical fractures of the ulna.

## 1. Introduction

An atypical fracture has been defined as a clinical condition that is highly associated with the long-term use of bisphosphonates [1] and occurs in the subtrochanteric region or femoral shaft [2]. In contrast, atypical fractures of the upper extremities are rare, since only 11 reports (15 fractures in 13 patients) have been published [3–13] (Table 1). Such fractures have a risk of nonunion; thus, accurate diagnosis and treatment are important. Here, we describe an atypical fracture of the ulnar diaphysis.

## 2. Case Presentation

A 78-year-old woman presented after falling from an upright position on her left hand and then experiencing difficulty elevating her left arm. She had a six-month history of mild pain in her left elbow after agricultural work for which she used a hoe and a 10-year history of medication with bisphosphonate (alendronate), to treat osteoporosis that was diagnosed after a thoracic vertebral fracture.

**Figure 1:**
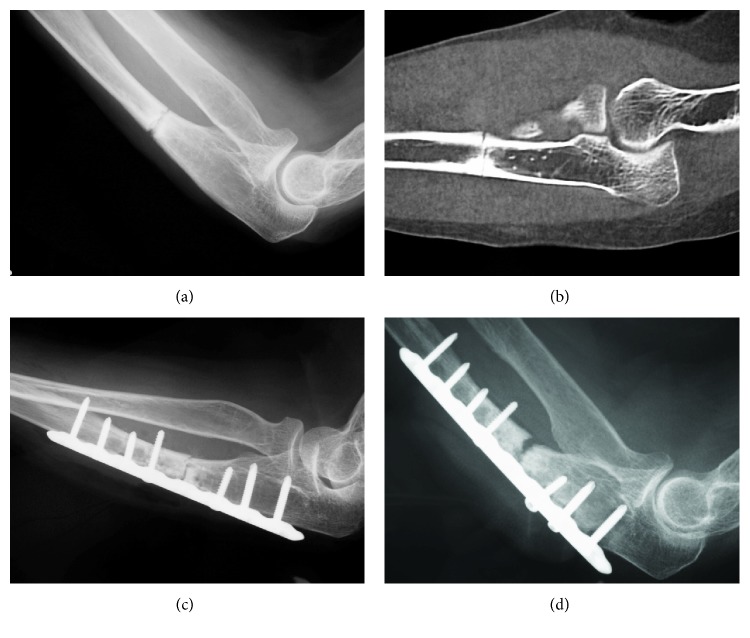
Imaging findings. Plain radiography (a) and computed tomography (b) images show transverse fracture at the proximal ulna. Plain radiographs immediately after open reduction with internal fixation (c) and at three months thereafter (d).

**Figure 2:**
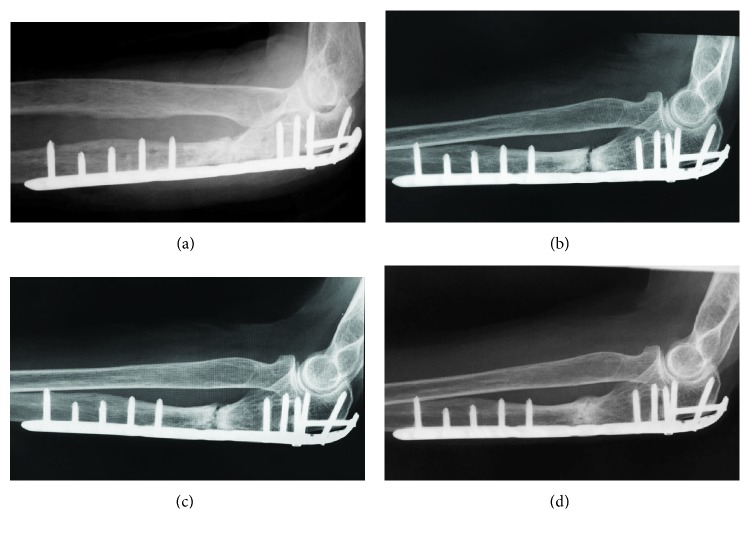
Findings of plain radiography after reoperation. Immediately after the second operation (a). Two months after the second operation, bone absorption is evident (b). The gap at the fracture site has decreased in 19 months after reoperation (c), and bone union is complete at 30 months after reoperation (d).

A lateral radiograph of the left forearm upon presentation revealed a transverse fracture at the posterolateral aspect of the proximal ulna (Figure 1(a)). Computed tomography of the left ulna showed cortical thickening, with transverse fractures and spike formation on the volar side (Figure 1(b)). X-rays did not reveal any abnormalities of the contralateral side. Biochemical findings showed normal serum calcium, phosphate, alkaline phosphatase, and thyroid hormone values. The mineral density of the distal radius had a *T* score with a standard deviation (SD) of -1.9 on dual-energy X-ray absorptiometry. The fracture was diagnosed as atypical, and bisphosphonate therapy was stopped. The patient underwent open reduction with internal fixation (ORIF) using a 3.5 mm locking compression plate (DePuy Synthes, Zeist, Netherlands) for the ulnar fracture (Figure 1(c)). A transverse fracture with cortical thickening was evident during the procedure. Plain radiography showed no signs of fracture healing at three months postoperatively (Figure 1(d)), when the patient complained of pain at the fracture site. The results of a physical examination and laboratory tests, including white blood cell counts, C-reactive protein, and erythrocyte sedimentation rate, were normal, which ruled out an underlying infection of the surgical site. We diagnosed the fracture site as nonunion; thus, we performed the revision surgery to stabilize and to achieve a union of the fracture site at 4 months after the first operation. Scar tissue and the osteosclerotic lesion from the fracture site were excised under general anesthesia, and the fracture surfaces were freshened. An autologous corticocancellous bone graft from the iliac crest was inserted at the resection site, and osteosynthesis was proceeded using a locking plate indicated for olecranon fractures (DePuy Synthes, Zeist, Netherlands) (Figure 2(a)). Immediately after this procedure, low-intensity pulsed ultrasound (LIPUS) (SAFHS; Teijin Pharma, Tokyo, Japan) was applied once a day for 20 minutes. However, a callus was not observed, and bony absorption was visible at the bone graft site at two months after the reoperation (Figure 2(b)). Therefore, the patient consented to empirical, off-label therapy with teriparatide at doses approved to treat osteoporosis (20 *μ*g/day). After 19 months on teriparatide, X-rays revealed bone bridges and a decreased gap between the fragments (Figure 2(c)). Healing after 30 months of treatment with LIPUS and teriparatide was complete (Figure 2(d)), and this coincided with the disappearance of pain and a complete range of elbow and wrist joint motion.

## 3. Discussion

A relationship between long-term bisphosphonate therapy and atypical femoral fractures has been suggested [1, 2], and these conditions might be closely related to severely suppressed bone turnover (SSBT) [14]. In contrast, only 11 reports in the English literature have described atypical fractures of the upper extremities [3–13], and they usually developed in the absence of traumatic events or as a result of minimal trauma (Table 1). Our patient used a hoe daily and sustained a fall from an upright position onto her left hand. One report has described that a transverse configuration, localized periosteal, or endosteal thickening at the fracture site and generalized cortical thickening of the diaphysis are features of atypical ulnar fractures [15]. Some authors have postulated that the mechanism is related to SSBT because of pathological findings [7], whereas others have speculated that the mechanism is related to cyclical weight-bearing during ambulation while walking with a cane or frame [6, 9, 10, 13]. The injured forearm of our patient had some features of atypical fractures on radiography images, she had been under long-term bisphosphonate therapy, and she habitually used a hoe during agricultural work. We diagnosed an atypical fracture of the ulnar diaphysis based on these features, which were associated with a stress mechanism.

Atypical femoral fractures are often difficult to manage because of delayed union and a high incidence of revision surgery [16]. Teriparatide as monotherapy and in combination with calcium and vitamin D exerts osteosynthetic effects on atypical femoral fractures associated with bisphosphonates [17–19]. In contrast, the management of rare atypical fractures of the upper extremities has not been established. Four patients have been treated conservatively [4, 6, 10, 13], and four others had fractures fixed with a plate and a bone graft [5, 7, 12]. Four of five fractures treated by internal rigid fixation [3, 8, 9, 11] united uneventfully, and complications developed in one patient during the healing process.

The effects of teriparatide on healing atypical femoral fractures have been assessed [17–19], but teriparatide has only been applied to atypical fractures of the upper extremities of two patients [12]. One recent patient required teriparatide because callus was not evident and bony absorption was visible at the bone graft site after reoperation. Considering all these findings, we believe that atypical ulnar fractures could be treated by internal rigid fixation with a bone graft under teriparatide and LIPUS. However, we think that evidences of LIPUS use for atypical fractures are still insufficient. Further investigations are needed to confirm the beneficial effects of LIPUS use in addition to the teriparatide for atypical fractures.

The present study has some limitations. Because we did not collect a bone biopsy, we could not histologically rule out SSBT, and the patient cohort was small. Further larger studies with complete bone metabolic markers, histological specimens, X-ray evidence, an appropriate surgical procedure, and combined therapy such as teriparatide and LIPUS are needed to confirm our findings.

In conclusion, although an atypical fracture of the ulna is rare, long-term medication with bisphosphonate and typical findings of fracture on images can help to conclude a diagnosis. An atypical fracture of the ulna can be treated with careful preoperative planning and postoperative therapy with LIPUS and teriparatide.

## Figures and Tables

**Table 1:** Reports of atypical ulnar fractures.

Authors	Age (y)	Sex	Affected side	Cause/trauma	Bisphosphonate history	Treatment	Outcome	Remarks
Moon et al. (2013)	76	F	Left	NHT	Alendronate	Internal fixation	Union	
	78	F	Left	NHT	Alendronate	Conservative	Some healing at 3 m	
Stathopoulos et al. [3]	76	F	Right	NHT	Zoledronate	Internal fixation	Union	
Tang and Kumar [11]	7	F	Right	NHT	Alendronate	Conservative	Nonunion	
Bjørgul and Reigstad [5]	83	F	Left	Crutch use	Alendronate	Internal fixation with bone graft	Union	
Ang et al. [6]	84	M	Bilateral	Walking frame use	Alendronate	Conservative	NS	
Chiang et al. [9]	77	F	Right	Walking cane use	Alendronate	Internal fixation	Nonunion, managed with bone graft and refixation	
Osada et al. [7]	85	F	Left	Light fall	Alendronate	Internal fixation with bone graft	Union	
Erdem et al. [10]	62	F	Right	Walking cane use	Alendronate	Conservative	Nonunion	
Shimada et al. [12]	79	F	Right	NHT	Alendronate	Internal fixation with bone graft	Union	Teriparatide+LIPUS
	89	F	Left	Lightly hit elbow	Risedronate	Internal fixation with bone graft	Union	Teriparatide+LIPUS
Yam and Kwek [13]	89	F	Bilateral	Walking frame use and fall	Alendronate	Conservative	Non-union	
Oh et al. [11]	72	F	Left	Light fall	Alendronate	Internal fixation	Union	
Present study	78	F	Left	Hoe use and fall	Alendronate	Internal fixation	Nonunion, managed with bone graft and refixation	Teriparatide+LIPUS

LIPUS: low-intensity pulsed ultrasound; NHT: no history of trauma; NS: not stated.

## References

[1] Schilcher J., Koeppen V., Aspenberg P., Michaëlsson K. (2015). Risk of atypical femoral fracture during and after bisphosphonate use. *Acta Orthopaedica*.

[2] Thompson R. N., Phillips J. R., McCauley S. H., Elliott J. R., Moran C. G. (2012). Atypical femoral fractures and bisphosphonate treatment: experience in two large United Kingdom teaching hospitals. *The Journal of Bone and Joint Surgery. British Volume*.

[3] Stathopoulos K. D., Kosmidis C., Lyritis G. P. (2011). Atypical fractures of the femur and ulna and complications of fracture healing in a 76-year-old woman with Sjögren’s syndrome. *Journal of Musculoskeletal and Neuronal Interactions*.

[4] Tang Z. H., Kumar V. P. (2011). Alendronate-associated ulnar and tibial fractures: a case report. *Journal of Orthopaedic Surgery (Hong Kong)*.

[5] Bjørgul K., Reigstad A. (2011). Atypical fracture of the ulna associated with alendronate use –a case report. *Acta Orthopaedica*.

[6] Ang B. F. H., Koh J. S. B., Ng A. C. M., Howe T. S. (2013). Bilateral ulna fractures associated with bisphosphonate therapy. *Osteoporosis International*.

[7] Osada R., Zukawa M., Kimura T. (2015). Atypical ulnar fracture associated with long-term bisphosphonate use. *Journal of Orthopaedic Science*.

[8] Moon J., Bither N., Lee T. (2013). Atypical forearm fractures associated with long-term use of bisphosphonate. *Archives of Orthopaedic and Trauma Surgery*.

[9] Chiang G. S., Koh K. W., Chong T. W., Tan B. Y. (2014). Stress fracture of the ulna associated with bisphosphonate therapy and use of walking aid. *Osteoporosis International*.

[10] Erdem Y., Atbasi Z., Emre T. Y., Kavadar G., Demiralp B. (2016). Effect of long-term use of bisphosphonates on forearm bone: atypical ulna fractures in elderly woman with osteoporosis. *Case Reports in Orthopedics*.

[11] Oh B. H., Heo Y. M., Yi J. W., Kim T. G., Lee J. S. (2018). Atypical fracture of the proximal shaft of the ulna associated with prolonged bisphosphonate therapy. *Clinics in Orthopedic Surgery*.

[12] Shimada Y., Ishikawa T., Endo J. (2017). Treatment of atypical ulnar fractures associated with long-term bisphosphonate therapy for osteoporosis: autogenous bone graft with internal fixation. *Case Reports in Orthopedics*.

[13] Yam M. G., Kwek E. B. (2017). A case of bilateral atypical ulnar fractures with bisphosphonate therapy in a walking aided elderly. *Annals of the Academy of Medicine, Singapore*.

[14] Visekruna M., Wilson D., McKiernan F. E. (2008). Severely suppressed bone turnover and atypical skeletal fragility. *The Journal of Clinical Endocrinology and Metabolism*.

[15] Tan S. H. S., Saseendar S., Tan B. H. M., Pawaskar A., Kumar V. P. (2015). Ulnar fractures with bisphosphonate therapy: a systematic review of published case reports. *Osteoporosis International*.

[16] Meling T., Nawab A., Harboe K., Fosse L. (2014). Atypical femoral fractures in elderly women. *The Bone & Joint Journal*.

[17] Carvalho N. N. C., Voss L. A., Almeida M. O. P., Salgado C. L., Bandeira F. (2011). Atypical femoral fractures during prolonged use of bisphosphonates: short-term responses to strontium ranelate and teriparatide. *The Journal of Clinical Endocrinology and Metabolism*.

[18] Unnanuntana A., Saleh A., Mensah K. A., Kleimeyer J. P., Lane J. M. (2013). Atypical femoral fractures: what do we know about them? AAOS Exhibit Selection. *The Journal of Bone and Joint Surgery-American Volume*.

[19] Cozzi Lepri A., Capone A., Del Prete A., Soderi S., Muncibi F., Civinini R. (2018). Atypical femur fractures. *Clinical Cases in Mineral and Bone Metabolism*.

